# Editorial: Regulation of endoplasmic reticulum and mitochondria in cellular homeostasis

**DOI:** 10.3389/fcell.2022.1004376

**Published:** 2022-08-30

**Authors:** Yongye Huang, Jianguang Ji, Qi Zhao, Jun Song

**Affiliations:** ^1^ College of Life and Health Sciences, Northeastern University, Shenyang, China; ^2^ Center for Primary Health Care Research, Department of Clinical Sciences, Lund University, Lund, Sweden; ^3^ School of Computer Science and Software Engineering, University of Science and Technology Liaoning, Anshan, China; ^4^ College of Life Science, Northeast Agriculture University, Harbin, China

**Keywords:** endoplasmic reticulum, mitochondria, homeostasis, redox, cell death, aging

## Hub: Endoplasmic reticulum centers in a variety of essential pathways

Endoplasmic reticulum (ER) is an interconnected single membrane-bound network and the largest organelle in the eukaryotic cell. The major function of ER includes the synthesis of proteins and lipids, protein folding and maturation, membrane biogenesis, xenobiotic detoxification, and cellular Ca^2+^ storage. Various mechanisms are conducted to help ER to complete these functions. ER-associated protein degradation (ERAD) mechanism is a conserved protein quality control pathway to erase misfolded, mistargeted and unassembled proteins to maintain ER homeostasis. ER stress, another critical mechanism to regulate ER homeostasis, is induced by the accumulation of misfolded proteins in the ER and triggers the unfolded protein response. Besides recovering cellular homeostasis to maintain cell survival, persistent or intense ER stress could also result in apoptosis, autophagic cell death, or ferroptosis. Altogether, targeting ER *via* modulating ERAD or ER stress would be beneficial for organism development and disease therapy.

## Powerhouse: Mitochondria serve as an energic and metabolic center

Mitochondrion is a complex double-membraned cellular organelle that harbors their own genome. Mitochondrion acts as the energy-producing center in cells for producing ATP *via* the oxidative phosphorylation system. The mitochondrion also serves as a powerful center for the precursors of macromolecules, such as DNA, RNA, proteins, and lipids. Besides the well-known roles in cell metabolism and energy conversion, mitochondria also participate in redox homeostasis, Ca^2+^ homeostasis, stress response, metabolite transport, cell signaling, cell cycle distribution, differentiation, cell death, embryonic development, and aging. Emerging evidence has shown that mitochondrial malfunction is closely related to a broad spectrum of human diseases, including cancer and neurological disorder. Mitochondria execute these functions *via* various molecular and cellular mechanisms. For example, mitophagy, a tightly regulated biological process defined as autophagy-dependent selective degradation of damaged or excessive mitochondria, acts an important role in the quality and quantity control of mitochondria to maintain ultimate cellular homeostasis. Mitochondria-mediated intrinsic apoptosis, regulated by caspases, Bcl-2 family of proteins, Smac/DIABLO, death receptors, IAPs, Omi/HtrA2 and cytochrome c, is one of the major apoptotic pathways. In sum, modulating mitochondria-mediated cell apoptosis, mitochondrial dynamics, mitochondrial energy metabolism, and some other mitochondrial physiological processes would be critical for cells to rebuild homeostasis.

## Offensive and defensive alliance: Crosstalk between endoplasmic reticulum and mitochondria

ER and mitochondria are all essential for maintaining cellular homeostasis. To fulfill the goal, an efficient and fast exchange of materials between the ER and mitochondria is required. In addition, there are physical interactions between ER and mitochondria at specific sites (named mitochondria-associated ER membrane, MAMs) in which the surfaces of the two organelles juxtapose at a constant distance, for several nm in length. The unique contact sites serve as the platform for communication between the two organelles and are critical for the regulation of cellular homeostasis, including Ca^2+^ homeostasis, proteostasis, and ER redox homeostasis. Different pathways and numerous mediators participate in the cross-talk between ER and mitochondria. For example, Mitofusin 2 (Mfn2), fission protein 1 homologue (Fis1) and B-cell receptor-associated protein 31 (BAP31) play a crucial role in mitochondrial fusion and fission (Barazzuol et al., 2021). Among them, BAP31, a polytopic integral membrane protein of ER, functions in apoptosis and participates in several kinds of diseases, including cancers (Li et al., 2022a). BAP31 interacting with Fis1 in MAMs generates an essential platform for the procaspase eight recruitment and transduces apoptotic signal from mitochondria to ER. BAP31 could be cleaved into proapoptotic p20BAP31 in case procaspase eight is recruited and activated to transmit ER calcium signals to mitochondria rapidly. Certainly, there are also some other proteins interacting in MAMs to bridge the crosstalk between ER and mitochondria.

## Bumper harvest and future perspective

In this Research Topic, we have published 14 papers. Excitedly, there are many novel findings regarding the mechanism and/or application associated with ER and mitochondria. 5-hydroxymethylfurfural, a furan-containing aldehyde widely presented in various sacchariferous foods, is shown to alleviate inflammatory lung injury by suppressing ER stress (Zhang et al.). In another study, a lipophilic glycoprotein named milk fat globule EGF factor 8 (MFG-E8) is reported to inhibit ER stress to maintain cellular homeostasis in pancreatic exocrine acinar cells during acute pancreatitis (Ren et al.). These studies convey a message that ER stress acts as an important mediator in the various physiological and pathological processes. In our previous studies, we also found that modulating ER stress could be beneficial for inhibiting cancer development (Huang et al., 2021; Li et al., 2022b). Since the existence of the ER-mitochondria interface, the stress from ER could also send a signal to mitochondria and even further receive feedback from mitochondria. In this Research Topic, Rajalekshmy Shyam etc. report that there was dilated ER and elevated expression of ER stress markers BIP and CHOP in mouse Slc4a11^−/−^ corneal endothelial tissue, and concludes that mitochondrial ROS could induce ER stress (Shyam et al.). Furthermore, the Research Topic also collected several review articles deciphering the role of ER and mitochondria in many biological events.

**FIGURE 1:**
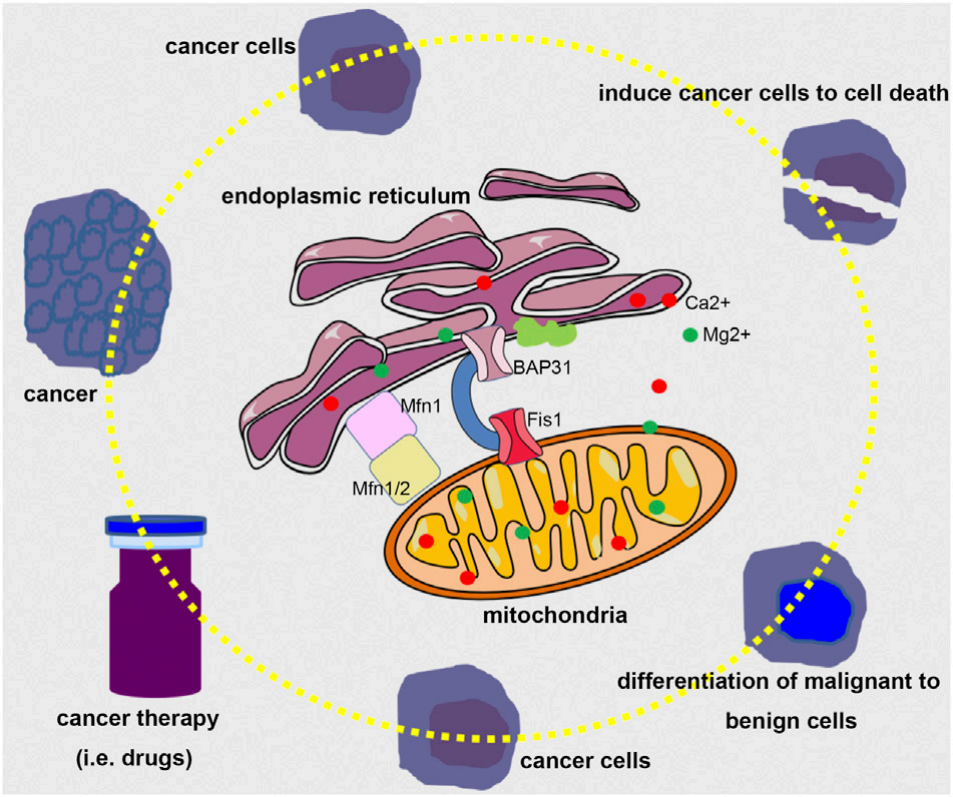
A bold assumption for modulating the crosstalk between ER and mitochondria in cancer therapy. Considering the significant role of ER and mitochondria in determining cells to restore cellular homeostasis or induce programmed cell death, it would be possible that tuning the interplay between ER and mitochondria using proper therapeutic means could guide cancer cells to death or differentiate malignant to benign cells.

However, it is a pity that we just received limited papers about the mechanisms of ER and mitochondria regulating cellular homeostasis, the contribution of modulating these two organelles to embryo and disease development, and novel methods to assess the crosstalk between ER and mitochondria. In addition, we consider that it would be wonderful to investigate the crosstalk between ER and mitochondria in future research using editing techniques, such as CRISPR-Cas9 and DdCBE. It would also be interesting to study the molecular mechanism between ER and mitochondria interplay and novel cell death modes, such as ferroptosis, NETosis, pyroptosis, and cuproptosis. Expectedly, it would have great significance for regulating the ER and mitochondria to maintain cellular homeostasis to prevent the occurrence of disease (Figure 1).
